# Comprehensive pulmonary rehabilitation in home-based online groups: a mixed method pilot study in COPD

**DOI:** 10.1186/s13104-015-1713-8

**Published:** 2015-12-10

**Authors:** Tatjana M. Burkow, Lars K. Vognild, Elin Johnsen, Marijke Jongsma Risberg, Astrid Bratvold, Elin Breivik, Trine Krogstad, Audhild Hjalmarsen

**Affiliations:** Norwegian Centre for Integrated Care and Telemedicine, University Hospital of North Norway, P.O. Box 35, 9038 Tromsø, Norway; Norut, P.O. Box 6434, Forskningsparken, 9294 Tromsø, Norway; University Hospital of North Norway, P.O. Box 100, 9291 Tromsø, Norway; Helse Nord FIKS, Northern Norway Regional Health Authority, Tromsø, Norway; Department of Clinical Medicine, UiT The Arctic University of Norway, 9037 Tromsø, Norway

**Keywords:** Pulmonary rehabilitation, Home-based, Group-based, COPD, Internet, Programme costs

## Abstract

**Background:**

Comprehensive multidisciplinary pulmonary rehabilitation is vital in the management of chronic obstructive pulmonary disease (COPD) and is considered for any stage of the disease. Rehabilitation programmes are often centre-based and organised in groups. However, the distance from the patient’s home to the centre and lack of transportation may hinder participation. Rehabilitation at home can improve access to care for patients regardless of disease severity. We had previously studied the technology usability and acceptability of a comprehensive home rehabilitation programme designed for patients with very severe COPD receiving long-term oxygen therapy. The acceptability of such comprehensive home programmes for those with less severe COPD, who may be less homebound, is not known. The aims of this feasibility study were to assess patient acceptability of the delivery mode and components of a comprehensive pulmonary rehabilitation programme for any stage of COPD, as well as the technology usability, patient outcomes and economic aspects.

**Methods:**

Ten participants with COPD in the Global Initiative for Chronic Obstructive Lung Disease (GOLD) grade I–IV were enrolled in a 9-week home programme and divided into two rehabilitation groups, with five patients in each group. The programme included exercise training and self-management education in online groups of patients, and individual online consultations. The patients also kept a digital health diary. To assess the acceptability of the programme, the patients were interviewed after the intervention using a semi-structured interview guide. In addition the number of sessions attended was observed. The usability of the technology was assessed using interviews and the System Usability Scale questionnaire. The St George’s Respiratory Questionnaire (SGRQ) was used to measure health-related quality of life.

**Results:**

The mode of delivery and the components of the programme were well accepted by the patients. The programme provided an environment for learning from both healthcare professionals and peers, for asking questions and discussing disease-related issues and for group exercising. The patients considered that it facilitated health-enhancing behaviours and social interactions with a social group formed among the participants. Even participants who were potentially less homebound appreciated the home group and social aspects of the programme. The participants found the technology easy to learn and use. The acceptability and usability results were consistent with those in our previous study of patients with very severe COPD. Only the mean change in the SGRQ total score of −6.53 (CI 95 % −0.38 to −12.68, *p* = 0.04) indicates a probable clinically significant effect. Economic calculations indicated that the cost of the programme was feasible.

**Conclusions:**

The results of this study indicate that comprehensive pulmonary rehabilitation delivered in home-based online groups may be feasible in COPD. The mode of delivery and components of the programme appeared to be acceptable across patients with different disease severity. The results in terms of patient outcomes are inconclusive, and further assessment is needed.

## Background

Chronic obstructive pulmonary disease (COPD) is incurable, and its prevalence is increasing [1, 2]. The disease leads to impairments, such as a reduced quality of life and exercise capacity. Pulmonary rehabilitation is vital in the management of COPD [3–7] and is considered for any stage of the disease. Patient outcomes include improved health-related quality of life (HRQL), reduced dyspnoea and increased exercise capacity [5]. The 2013 American Thoracic Society and European Respiratory Society statement [4] defines pulmonary rehabilitation as a comprehensive intervention “based on a thorough patient assessment followed by patient-tailored therapies, which include, but are not limited to, exercise training, education, and behaviour change, designed to improve the physical and psychological condition of people with chronic respiratory disease and to promote the long-term adherence of health-enhancing behaviours”. Pulmonary rehabilitation can be offered on both an inpatient and an outpatient basis, and the typical programme duration is 6–12 weeks [6]. Programmes are often organised in group settings [3] because groups can be helpful with respect to learning and sharing experiences [4].

The travelling distance from the patient’s home to the rehabilitation venue, lack of transportation, restricted physical mobility of patients and inability to travel independently may hinder uptake and completion of centre-based programmes [8, 9]. Rehabilitation provided in the patient’s home could provide easier access to programmes. However, lack of group support may be a potential drawback in the home setting [3]. Internet-enabled programmes may help overcome this problem.

There are few studies on comprehensive pulmonary rehabilitation in group settings at home. Nguyen et al. [10] studied a dyspnoea self-management programme for COPD with logging of symptoms and exercise data, access to educational material, and the possibility of peer interaction in chat sessions. The study was terminated earlier than planned due to technical challenges, but their results showed reduced dyspnoea in activities of daily living. Taylor et al. [11, 12] found in two small studies that home group exercise and advice sessions for COPD supervised by a physiotherapist using videoconferencing were well received by the participants. Videoconferencing has also been used to deliver group education to COPD patients in remote centres, combined with group exercise training under local supervision [13]. We are not aware of videoconferencing being used for group education to COPD patients in the home setting. However, there are several studies on exercise training for individual patients at home. Maltais et al. [14] report on a comprehensive programme where the exercise-training component was provided for individual patients at home, while the self-management education was given in an outpatient group setting at a hospital. Minet et al. [15] used videoconferencing for delivering individual home-based exercise training and counselling after hospitalization. There are also other studies on technology-assisted exercise training for individual patients in the home setting, including those using mobile devices such as cell phones [16, 17].

We had previously assessed patient acceptability and technology usability of an Internet-based comprehensive, multidisciplinary pulmonary rehabilitation programme in home groups for patients with very severe COPD who were receiving long-term oxygen therapy (LTOT) [18, 19]. Disease severity, especially LTOT use, has been associated with poor uptake and completion of centre-based pulmonary rehabilitation [20]. The previous home programme included exercise training and self-management education in online groups of patients, and individual online consultations. The patients also kept a digital health diary to be reviewed in the consultations. For practical reasons, the home programme lasted no longer than 6 weeks. Five patients were enrolled in one online rehabilitation group. The patients found the technology easy to use and they were highly accepting of the programme and its components.

The question remained whether patients who were more mobile and less burdened by their disease would find home pulmonary rehabilitation equally acceptable. We therefore adapted the previous home programme to patients with different levels of COPD severity. The content of the digital health diary was adjusted for a broader disease spectrum, and a traffic-light colour code was used for visualisation of some of the content. The home programme was extended to 9 weeks in order to better resemble the hospital outpatient programme on which the home programmes were based, and therefore covered more education topics and exercise training sessions than the previous 6-week home programme. The patients were also given a step counter to see if it could motivate walking.

The aims of this feasibility study were to extend our previous investigation by assessing patient acceptability of the delivery mode and components of a comprehensive pulmonary rehabilitation programme for any stage of the disease, as well as the technology usability, patient outcomes and economic aspects.

## Methods

### Study design

This was a mixed-method pilot study. The patients were assessed at baseline, and assessed and interviewed shortly after the intervention. One outpatient rehabilitation clinic at a university hospital took part. The patients participated in a 9-week Internet-enabled home pulmonary rehabilitation programme. Ten patients were to be recruited, and enrolled in two rehabilitation groups. The Regional Committee for Medical and Health Research Ethics (REC North) approved the study. The participants all provided written informed consent.

### Patient selection

The inclusion criteria for the study were a clinical diagnosis of COPD, age above 40, living in the county of Troms in northern Norway and previous participation in rehabilitation. Living in a location without potential access to a broadband network was an exclusion criterion. Healthcare personnel at the hospital’s outpatient rehabilitation clinic recruited the participants by sending a letter of invitation by postal mail to potential eligible patients.

### Data collection and analysis

#### Acceptability

Patient acceptability was defined as the extent to which services were generally approved and used, patients’ satisfaction with services and the perceived usefulness of services. Interviews were used to assess these different aspects of patient acceptability. In addition the number of sessions attended was observed.

A semi-structured interview guide with open-ended and closed questions was developed. The primary themes of the interview guide were user perceptions of the delivery mode and components of the programme. Shortly after the intervention, telephone interviews lasting about 1 h were conducted. One author structured the interview material, and three authors analysed it. For the analysis we applied what Lamont [21] denotes a theme-centred approach, and we performed what Maxwell [22] names a “descriptive interpretation”. The data were categorised and sorted into the study’s main themes. Thereafter an issue-focused [23], cross-case [24] analysis was performed where positive and negative aspects that the participants had responded to or mentioned were extracted and compared. Quotes from the interviews have been translated into English.

#### Technology usability

Technology usability was assessed using interviews and the System Usability Scale (SUS) [25, 26]. The SUS is an instrument for subjective assessment of technology usability, covering aspects such as technical training, complexity and the need for support. It is a 10-item questionnaire, which uses a five-point Likert scale. Scores range from 0 to 100, where 100 is the best score. The SUS was administered post-programme, and mean and median values were calculated.

#### HRQL

The St George’s Respiratory Questionnaire (SGRQ) [27, 28] was used to measure the HRQL. The SGRQ has scores in three domains: symptoms, activity and impact, and a total score. Scores range from 0 to 100, with 100 indicating the worst quality of life impairment. The clinical threshold value for SGRQ in COPD on a group level is estimated to be a four-unit reduction [29]. Patients participating for less than 6 weeks were excluded from the outcome analysis, as 6–12 weeks’ participation has been shown to produce benefits in several patient outcomes [6].

#### Statistical analysis

For the SGRQ median and 25th percentile–75th percentile, mean and confidence interval were calculated. The *p* values were calculated using paired *t* test and z-value of 2.306, and *p* value of <0.05 was considered significant.

### Economic aspects

The costs of implementing the home programme in routine service by the Regional Health Authority of Northern Norway were compared to the Diagnosis-Related Group (DRG) reimbursement for outpatient rehabilitation.

Costs included were related to personnel salaries, travel, equipment, shipment of equipment and technical support. The salaries of healthcare personnel were calculated by using the average salary for each personnel group, including additional employers’ costs, such as social security premiums. Overhead costs of 10 % were added for preparatory work, administration, rent, electricity, etc. A 3-year lifetime was assumed for all investment items, and a discount rate of 4 % was used to calculate annual capital costs. A 10 % maintenance cost was included. It was assumed that the equipment could be reused for four home programmes per year. Investments in necessary equipment were estimated based on market prices. The need for technical support and training was estimated based on data from the pilot. Travel costs for participants were estimated based on the average travel distances for the participants in this pilot assuming the use of a private car. It was assumed that participants had a broadband connection at home, and that videoconference studios at a hospital could be used for the videoconference sessions, at no extra cost.

### Organisation and content of the home programme

The home programme was a modified version of the programme described in [19], and both were based on the outpatient pulmonary rehabilitation programme at the University Hospital of North Norway. It included weekly group-based education and exercising, as well as individual consultations, for 9 weeks at home. The participants in a rehabilitation group enrolled at the same time.

A multidisciplinary team consisting of a specialist, nurse, physiotherapist, nutritionist and social worker provided the online group education sessions. The education sessions were held once a week, lasting 60 min, and in a lecture and discussion format.

Table 1 shows the education topics covered in the programme. For some topics, the patients were requested to watch one or more online videos before the sessions. The videos were not of TV-broadcast quality and featured the healthcare professionals in the team.

**Table 1:** Educational session topics

Proper use of medications Breathing strategies, anxiety and panic control, relaxation techniques and stress management Energy conservation Pathophysiology of lung disease Benefits and maintenance of physical activity Prevention and early treatment of respiratory exacerbations Nutrition Social security rights Maintaining the benefits of educational and exercise training	

A physiotherapist led the online group exercise sessions. There were two sessions each week, each lasting 30 min. Each session involved 5–10 min of warming up before starting with strength and endurance exercises for upper and lower extremities using constant load, as well as interval training. The intensity allowed for all to be able to participate. Sticks were used to emphasize thorax mobilization and elastic bands to provide resistance for strength training. The training programme was somewhat intensified for each session. Both low- and high-intensity exercise training have shown benefits for patients with COPD [6]. For additional exercise training during the week, the participants were asked to use an online follow-along exercise video at least once or twice a week. The participants were also given a simple and easy-to-use step counter (Yamax SW200 Digi-Walker Pedometer) to determine whether counting steps would motivate walking.

The online individual consultations were with a nurse or a physiotherapist and held once a week lasting 10–15 min. The patients kept a digital diary of health questions (Table 2), pulse oximetry (SpO_2_) values and step counts, which were reviewed in the individual consultations. An in-person meeting was held for each rehabilitation group at the hospital rehabilitation clinic before and after the home programme. Tables 3 and 4 provide a more detailed overview of the programme plan.

**Table 2:** Daily questions in the digital health diary

How was your day (worse than normal, normal, better than normal)? Have you been outside your home today (less than normal, normal, more than normal)? How many meals have you had today (two to three, five, more than five)? Did you have breathing problems today (worse than normal, normal, better than normal)? Have you been bothered by coughing today (worse than normal, normal, better than normal)? Amount of sputum^a^ (less than normal, normal, more than normal)? Colour of sputum^a^ (clear, coloured)?	

^a^Only asked if coughing worse than normal

**Table 3:** Pre- and post-programme in-person meetings

Meeting	Content
Pre home programme	Introduction
	Demonstration of the technology
	A group exercise session
	Assessment of each patient’s baseline
Post home programme	Assessment of each patient

**Table 4:** Week plan for the home rehabilitation programme

Frequency	Activity
Daily	Answer the daily questions in the digital health diary
	Measure and enter SpO_2_ values and accumulated step counts in the digital health diary
At least once or twice	Exercising with the follow-along exercise video
Any day	Watch the educational video(s), if any, on the upcoming topic (10–40 min)
Tuesday	On-line group exercising session (30 min)
	A short break
	Online group education session in lecture and discussion format (60 min)
Friday	On-line group exercising session (30 min)
	Online individual consultation (approximately 10–15 min each)

### Technology

The prototype technology was a further development of the prototype used in the previous study [18, 19, 30]. At home, the user’s TV was connected to a small computer with an Internet connection, and a camera and a headset were used during videoconferencing. The participants used a numerical keypad on a remote control device to navigate the menus, enter user data and answer multiple-choice questions in the health diary. Pulse oximetry values and step counts were entered manually and displayed in graphs. The rest of the health diary data were visualised on the TV using a traffic-light colour code, with yellow indicating normal, red worse than normal and green better than normal. The videoconferencing multi-point control unit supported a maximum of six simultaneous participant sites, i.e. five home patients and one healthcare site. During multiparty videoconferencing, the TV screen was divided into six areas, one large and five smaller. The participants could see the educator or exercise instructor as well as the other participants on the screen. The largest area of the screen was given to the active speaker (either the educator or a patient) in the education sessions and to the physiotherapist in the exercise sessions. The healthcare personnel at the outpatient rehabilitation clinic used a stand-alone videoconferencing system for the group sessions and a PC for the individual video consultations.

Taking part in videoconference sessions in a living room raises issues of privacy, especially as family members or visitors present in the room can listen in on the discussions [31]. Therefore, if others were to be present, this had to be agreed upon in advance, and they had to be visible in the camera view. The participants were told that they could close the cover on the camera to safeguard their privacy when they were not taking part in the programme.

### Technical training and support

The patients were given a user manual and a technical training session during deployment of the equipment at home. They could call technical support staff during the programme period. Before the home programme started, a videoconferencing test session was held for each rehabilitation group. The healthcare personnel who had participated in the previous study received no technical training. A physiotherapist new to the home programme received a short technical training session before guiding the home exercise sessions. Technical support was available for the healthcare personnel during the videoconferencing sessions.

## Results

### Participants

Ten patients were recruited. According to the criteria of the Global Initiative for Chronic Obstructive Lung Disease (GOLD) [32], one participant was in grade I (mild), one in grade II (moderate), four in grade III (severe) and four in grade IV (very severe). Three were receiving long-term oxygen therapy, and one was using oxygen only at night. The four participants in GOLD grade IV were more homebound than the other participants. Homebound patients find it difficult to leave home and typically do not do so due to the burden of their disease. The average age was 61.7 (range 46–72; median 60). Five participants were male and five were female. Two were working, and the rest were retired. Eight of the ten patients lived in a one-person household. Two had participated in our previous study [19]. Two of the participants had used computer systems in their jobs but not after retirement. The remaining eight participants all used computers, with one starting after hearing about the study. Seven of the participants used the Internet, and four of these used email.

Table 5 summarises the baseline characteristics of the participants.

**Table 5:** Characteristics of the participants

	n = 10
Gender	
Male	5
Female	5
Age (years)	
45–54	1
55–64	6
65–74	3
Employment status	
Working	2
Retired	8
Distance from outpatient clinic (km)	
0.5–20	3
50–110	4
180–220	3
GOLD grade	
I	1
II	1
III	4
IV	4
LTOT users	3
FEV_1_ % mean (SD)	40.3 (24.48)
FEV_1_/FVC mean (SD)	47.6 (13.33)

The ten participants were enrolled in two rehabilitation groups, with five in each. One group (P1–P5) followed the complete programme plan (Tables 3, 4). Participants in the other group (P6–P10) did not meet each other in person before and after the home programme due to severe impairments, combined with long travelling distances for some of the participants. For practical reasons, this group exercised together only once a week. The rest of their programme was according to the programme plan. Two different physiotherapists led the exercise sessions in the two groups. The first group participated from October to December 2007, and the second group from February to April 2008. The healthcare team consisted of five women and one man, of whom four had participated in our previous study [19].

### Acceptability

#### General approval

The online home programme was well perceived by the participants, illustrated by quotes such as “really good” (P1), “wonderful” (P5) and “outstanding” (P7). They also described it as follows:

… fantastic for those of us who live so far away from the hospital… I have to get up in the middle of the night if I have to do anything in town, and I don’t get home until the evening. (P4)

I think it’s absolutely great because I’m so ill that it’s a real strain to go out. (P9)

Otherwise, there is nothing. You have to travel away from here to get anything, physiotherapy, or exercise or things like that. So, I think this is wonderful. (P10)

All the participants said they would recommend the programme to others, as expressed by one: “Yes, absolutely.” (P2).

#### Attendance of online sessions

There was high attendance of the online sessions. Eight patients attended all group and individual sessions, one patient once missed the Tuesday group sessions, while the tenth patient participated for <6 weeks due to hospital admissions.

#### Group education

The online group education created an opportunity for participants to learn from the healthcare personnel and to ask questions. It was well received. The participants found it useful both for learning new information and for refreshing knowledge:

As I mentioned, I learnt a lot… And it was great to have the opportunity to ask questions. (P4)

It was very informative. Got answers to a lot that I was not sure about. (P5)

it was… good to be reminded of things. Yes, so it was useful. (P10)

The possibility to share experiences and discuss issues with the other participants was appreciated. The participants learned from each other. Therefore, the other participants also became a source of information, supplementing the healthcare personnel.

It was as though we were sitting at the table over there and talking. So, it went very well… It is very good to meet others with the same illness and hear how they experience it……. even if the disease is maybe not quite the same. (P3)

We learned from each other too… It helps to hear that other people are going through the same thing. That they struggle with things… Yes, how they do it… how they handle it. (P4)

Then you can exchange experiences and share advice. (P9)

They discussed many aspects related to the disease, such as its impact on daily life and how to live with the disease. Structure is needed for turn taking in videoconferencing, but the participants learned to adapt. All the participants watched the thematic educational video prior to the relevant education session.

#### Supervised group exercising, exercise video and step-counting

The online group exercising supervised by the physiotherapist worked well. It was fun and was well received:

The group exercise was fun. (P3)

And the exercise… I thought that was very important… it was great fun. (P7)

It was almost like being in the gym. (P10)

Exercising together and with a fixed schedule was perceived as motivating. The fact that others took part and that they could see each other encouraged the participants to make a greater effort:

It’s worse when you’re sitting there all alone … shall I do it tomorrow? You keep putting it off. But now you knew there was an exercise session, and you got ready and did the exercise… (P6)

For sure, when you have someone looking at you, you do what you are told. It’s worse if you are alone, then it’s easier to say – no, for heaven’s sake, today I’m so tired that I think I’ll take a break. But when it is full of people looking at you, then you do at bit more. (P9)

One participant expressed a wish to do additional exercising using gym equipment. All the participants used the follow-along exercise video, and some used it daily or nearly every day.

Five of the six participants in GOLD grade III and lower found using the step counter motivating, as expressed by one of them:

The step counter - that was a very motivating factor. Because, when you had sat there and been lazy one day, you felt ashamed to write the figures [in the diary]. (laughs) (P2)

The four participants in GOLD grade IV who were more homebound found the step counter of varying usefulness, but they all tried it. Two of them used the step counter while walking indoors, the third was walking too gently indoors for the step counter to detect, and the fourth preferred biking on his home stationary bicycle and logging kilometres instead of steps in the health diary.

#### Individual consultations and the digital health diary

The participants also perceived the online individual consultations as a valuable part of the programme:

But talking to XX… and being able to describe my situation – that’s also very important. (P1)

After all, you always have some specific questions that it’s good to be able to ask. (P8)

However, the need varied, depending on the stability of the illness. The diary was used as supplementary information and reviewed in the individual consultations. The participants updated the health diary. Most entered data on a daily basis, and two entered data for several days at a time. However, only a few looked at their data in the diary retrospectively.

#### Comprehensive programme

The comprehensive programme helped the participants by facilitating health-enhancing behaviours and integrating disease-related needs into daily life:

This is useful, perhaps especially because of what you learn… how you should take the medicine and learn that it doesn’t work right away. (P2)

There were many small things which are big things for me. Getting support. Motivation. I am not so afraid of doing things. I am exercising too. (P5)

I have learnt many little things… I became more secure and confident. (P6)

#### Supportive social environment

Several of the participants spontaneously emphasised the social contact and the social group that formed as a result of the programme, and the cohesion, openness and familiarity in the group.

It was as though we had known each other for a long time… it was really strange… The whole thing became very sociable too, because at the beginning I wouldn’t have believed that. But it did, in fact. (P3)

Even though you were kind of far away, they were so close to you. In a way, they were here at home with me. (P6)

I don’t know how to express this – it was as though you knew them. It felt as though we belonged together in a way. It was as though I knew them, even though I was here on my own, in a way. (laughs) (P7)

#### Programme organisation

The programme organisation was well accepted, and the total programme duration was not perceived as too long. The duration and frequency of the education sessions and individual consultations were perceived as appropriate. Several of the participants wanted more group exercise sessions per week or longer sessions. No difference was found between the two groups’ perception of the programme that could be attributed to not meeting in person before meeting online.

#### Intrusiveness

The participants did not find the camera on top of the TV or the computer behind the TV in the living room intrusive.

It was not intrusive in any way. (P2)

However, most of the participants closed the cover on the camera when it was not in use.

No, no special [reason]… it was like a kind of habit you got used to when you were finished. (P8)

Yes, I used it. You can see when the light turns green. It shouldn’t be possible after all [to be monitored]. But. Might as well. (P9)

One participant elaborated on the challenge of using the TV in the living room when not living alone because the pulmonary rehabilitation session took place in the same shared living area.

### Technology usability

The participants found the technology easy to learn and use. This was illustrated by expressions, such as, “easy, not difficult” (P1), “it was simple” (P7) and “fine, straight away” (P8). They also noted:

I would never have believed it was possible to make something so simple. (P2)

So at the beginning I felt very afraid that I would not be able to do it, because I’m not very technical. But it was so easy that anybody at all could do it. (P3)

They found it easy to enter the data into the electronic diary and access the diary, access the education videos and use the videoconferencing system. They also found the traffic light and graph visualisation of the diary data easy to understand. The user manual was hardly needed at all, and most of the participants did not use it or read it only once. One of the participants experienced technical problems, mainly related to the wireless network connection. However, this did not seem to influence the acceptability of the programme for this participant.

Nine participants completed the SUS, with an average score of 94.4 (range 87.5–100, median 92.5).

### HRQL

The SGRQ scores in Table 6 show symptoms, impact, activity and total scores at baseline and post programme for the participants (*n* = 9). The SGRQ was administered in person, except for one group to which it was administered by telephone at baseline. One patient who participated for <6 weeks was excluded from the analysis.

**Table 6:** Change in SGRQ scores from baseline to post programme, with change given as mean and *p* value (using paired *t* test and z-value of 2.306)

SGRQ	Baseline	Post programme	Mean change [95 % CI]	*p* value
Symptoms	50.5 (21.6–78.9)	31.9 (24.7–48.2)	−8.61 [−23.32, 6.10]	0.21
Activity	59.5 (57.7–85.9)	60.4 (53.6–79.1)	−3.13 [−8.53, 2.27]	0.22
Impact	44.1 (40.7–57.9)	39.0 (34.8–43.9)	−6.79 [−14.10, 0.52]	0.06
Total	58.0 (42.7–60.7)	45.5 (35.6–61.0)	−6.53 [−12.68, −0.38]	0.04

Data are in median and (25th percentile–75th percentile)

### Economic aspects

The total cost of the home programme was estimated at €581 per participant (at 2012 prices). The cost components are shown in Table 7. The health care personnel provided 1 h of group education, 1 h of group exercise and 15 min per patient of individual consultations weekly for 9 weeks at home. Pre and post home programme in-person group meetings required 6 h of health personnel time in total. Personnel costs accounted for 65 % of the costs of this home rehabilitation programme, making the total cost sensitive to local pay levels. The calculations assumed five patients in each home group, and a larger group size would lower the costs per participant through lower personnel costs.

**Table 7:** Costs of home pulmonary rehabilitation programme per participant at 2012 prices

Cost component	Costs (€)
Healthcare personnel	355
Travel	120
Equipment	46
Transport (deployment)	35
Technical support	25
Total costs	581

Most health personnel participating in the trial were able to use the technology with minimal training. Therefore, the costs of personnel training were not included in these calculations. The need for technical support during the videoconference sessions was estimated at 15 min per week. The average travel distance for a participant attending in-person group meetings was estimated at 60 km (one way). If a videoconference studio at a hospital could not be used, multi-point control unit services for handling many-to-many communication would add a cost of €8 per month. For participants without a fixed broadband connection, a mobile Internet connection for 5 months would add a total cost of €75. An extra cost might be incurred in some cases if the participants needed professional support to install the equipment.

The economic calculations indicated that the cost per patient of the home programme was lower than the DRG reimbursement rate for outpatient rehabilitation (€1680 in 2012).

## Discussion

### Principal results

The mode of delivery and the components of the programme were well accepted by the patients, and the acceptance seemed to be independent of disease severity. The programme provided an environment for learning from healthcare professionals and from peers, for asking questions and discussing disease-related issues and for group exercising. The patients considered that it facilitated health-enhancing behaviours and social interactions, with a social group formed among the participants. Meeting in person before meeting online did not seem to influence the programme’s acceptability. This indicates that the programme could be organised without the in-person meetings and save burdensome travelling. Many of the participants would have preferred more group exercising, by increasing either the frequency or the duration. Both the interviews and the SUS scores indicate high usability of the technology in this context. The acceptability and usability results are consistent with those of our previous study of patients with very severe COPD [18, 19].

Only the change in the total SGRQ score of mean −6.53 (CI 95 % −0.38 to −12.68, *p* = 0.04) indicates a probable clinically significant effect. The improvement in the other SGRQ scores is promising, even though the differences are not significant.

The cost per patient of the home rehabilitation programme was lower than the reimbursement rate for outpatient rehabilitation.

### Limitations

This study was a pilot with a small sample size and no control group. The statistical analyses must be interpreted with great caution given the small sample, which may not be representative of the target population. To limit result bias, several researchers were involved in the development of the interview guide and the analysis. Exercise capacity was not assessed in this paper.

Using airflow limitation as measured by spirometry to categorise disease severity does not necessarily reflect the impact of the disease, and the GOLD statement in 2011 introduced additional measures to reflect impact and symptom severity [32]. However, all four participants in GOLD grade IV were more homebound than the six participants in the lower GOLD grades.

Familiarity with computers was not an inclusion criterion. However, all except one of the patients who participated turned out to be current or past computer users. This may hinder generalisation to patients not familiar with computers. However, our previous study with both users and non-users of computers also indicated high usability. The fact that two of the patients had taken part in our previous study may have had a positive result bias on patient acceptability. These two patients would have been familiar with videoconference sessions and might have interacted differently with the other patients. However, the two patients were in the same group and no difference in acceptability was found between them and the other participants or between the two groups. Their opinions regarding usability also did not diverge from those of the other eight participants. The recruitment of the patients by the same clinic that delivered the home pulmonary rehabilitation programme may have led to a result bias.

Most of the healthcare personnel involved had long-term experience in pulmonary rehabilitation, which may limit the applicability of the programme to clinics where the personnel have little experience. The videos featured the same healthcare professionals who delivered the home programme, and the effect of using videos with unknown professionals is not known. The healthcare professionals’ opinion on the home programme delivery was not assessed in this paper. The cost of the home programme was not compared to the actual cost of the outpatient rehabilitation programme. Nor is it known whether home rehabilitation would be covered by the DRG for outpatient rehabilitation. The organisational changes needed to deliver the programme on a regular basis were not assessed.

### Further work

Further work would include conducting a study with a larger sample size comparing the patient outcomes of the home pulmonary rehabilitation programme with a control group receiving conventional rehabilitation.

## Conclusions

The results of this study indicate that comprehensive multidisciplinary pulmonary rehabilitation delivered in home-based online groups may be feasible for patients with COPD. The mode of delivery and components of the programme appeared to be acceptable to patients with different levels of disease severity. The programme provided an environment for learning both from healthcare personnel and from peers, for group exercising and for social support. The results in terms of patient outcomes are inconclusive, and further assessment is needed.

## Abbreviations

COPD: chronic obstructive pulmonary disease
FEV_1_: forced expiratory volume (1 s)
FVC: forced vital capacity
GOLD: Global Initiative for Chronic Obstructive Lung Disease
HRQL: health related quality of life
LTOT: long-term oxygen therapy
SGRQ: St George’s Respiratory Questionnaire
SUS: System Usability Scale
SpO_2_: pulse oximetry
